# Service organization protocol for coping with undergraduate students’ psychological distress: a collective construction

**DOI:** 10.1590/0034-7167-2022-0535

**Published:** 2023-10-09

**Authors:** Tatiane Carolina Martins Machado Rodrigues, Guilherme Correa Barbosa, Vera Lúcia Pamplona Tonete

**Affiliations:** I Universidade Estadual Paulista Júlio de Mesquita Filho. Botucatu, São Paulo, Brazil

**Keywords:** Mental Health, Students, Protocol, University, Coping Strategies., Salud Mental, Estudiantes, Protocolo, Universidad, Estrategias de Afrontamiento., Saúde Mental, Estudantes, Protocolo, Universidade, Estratégias de Enfrentamento.

## Abstract

**Objective:**

to report on the experience of the elaboration process of a service organization protocol for coping with public undergraduate students’ psychological distress in the countryside of São Paulo.

**Method:**

experience report on protocol production, an action research product, carried out according to the health care and service organization protocol model, made possible by remote meetings with 33 professionals linked to the management and services of the university’s health and social assistance departments.

**Results:**

collective protocol production provided an opportunity for an institutional agreement on educational, therapeutic and support actions, to be developed in groups or individually with students, with provision for permanent education with civil servants.

**Final considerations:**

this experience made it possible to list specific actions to face undergraduate students’ psychological distress, bringing health professionals closer to those in management, promoting the exchange of concepts and practices to re-signify and transform the work developed.

## RESUMO

**Objetivo:**

relatar experiência sobre o processo de elaboração coletiva de protocolo de organização de serviço para enfrentamento do sofrimento psíquico de estudantes de universidade pública do interior paulista.

**Método:**

relato de experiência da produção do protocolo, produto de pesquisa-ação, realizada conforme modelo de protocolo de cuidado à saúde e de organização de serviço, viabilizada por encontros remotos com 33 profissionais vinculados à gestão e aos serviços dos departamentos de saúde e assistência social da universidade.

**Resultados:**

a produção coletiva do protocolo oportunizou a pactuação institucional de ações educativas, terapêuticas e de apoio, para serem desenvolvidas em grupo ou individualmente com os estudantes, sendo previstas as de educação permanente com os servidores.

**Considerações finais:**

esta experiência possibilitou elencar ações específicas para o enfrentamento do sofrimento psíquico de estudantes universitários, aproximando profissionais da assistência aos da gestão, promovendo intercâmbio de concepções e práticas para ressignificar e transformar o trabalho desenvolvido.

## RESUMEN

**Objetivo:**

relatar la experiencia del proceso de elaboración colectiva de un protocolo de organización de servicios para el enfrentamiento del malestar psicológico de estudiantes de una universidad pública del interior de São Paulo.

**Método:**

relato de experiencia sobre la elaboración del protocolo, producto de investigación-acción, realizado según el modelo de protocolo de organización asistencial y de servicios de salud, posibilitado por encuentros a distancia con 33 profesionales vinculados a la gestión y servicios de los departamentos de salud y asistencia sociales universitarios.

**Resultados:**

la producción colectiva del protocolo brindó oportunidad para un acuerdo institucional sobre acciones educativas, terapéuticas y de apoyo, a ser desarrolladas en grupo o individualmente con los estudiantes, con previsión de educación permanente con los servidores públicos.

**Consideraciones finales:**

esta experiencia permitió enumerar acciones específicas para enfrentar el sufrimiento psíquico de estudiantes universitarios, acercando a los profesionales de la salud a los profesionales de la gestión, promoviendo el intercambio de conceptos y prácticas para resignificar y transformar el trabajo desarrollado.

## INTRODUCTION

Entering higher education is associated with a new reality for undergraduate students, permeated by satisfaction with the possibility of achieving the intended professional training, but also marked by uncertainties and challenges in the face of a universe full of new demands, responsibilities and social ties^(1)^.

This beginning of academic life coincides with a period of human development characterized by great physical, psychic and social changes that, not adapted to the university context, can lead students to psychological distress^(2)^.

During training, other factors can increase the vulnerability of undergraduate students to psychological distress, such as the overload of student tasks combined with problems in time management, in organization of life and financial routine, in finding an adequate place to live and establishing an efficient support network^(3-4)^.

Psychological distress in undergraduate students around the world and, in Brazil, has become increasingly present, especially aggravated by broader individual, family and socioeconomic problems, implying harmful consequences to academic and personal development, including being considered an emerging public health problem^(5)^.

For an effective approach to this mental health problem, it is recommended to ensure that students have opportunities to experience mental health promotion actions in the university context and, when seeking assistance due to the possibility of psychological distress, they are welcomed in an accessible and comfortable environment with duly trained and problem-solving professionals^(6)^.

It is believed that the formation of networks, with links established between students, professors and administrative technicians, can contribute to the consolidation of a solidary and humanized culture of support, configuring a fundamental strategy to promote this population’s health and quality of life^(3)^. At the same time, it is believed that strategies specifically designed and institutionally validated for coping with psychological distress are relevant and should be guided by interprofessional and intersectoral work^(7)^.

## OBJECTIVE

To report an experience on the collective elaboration process of a service organization protocol for coping with psychological distress among students at a public university in the countryside of São Paulo.

## METHOD

### Study design

This is a report on the experience of developing a service organization protocol for coping with psychological distress in undergraduate students, proposed as a product of an undergraduate thesis of a professional master’s course in nursing, which consisted of action research carried out by first author of this article, under the guidance of two professors of the aforementioned course. Action research is defined as participatory research, with a focus on respondents, planned with a view to changes within the researched situation, whose feedback is relevant to expanding knowledge and strengthening conviction in the product in question^(8)^.

As a guideline for the elaboration of this report, the REDEQUATOR COnsolidated criteria for REporting Qualitative research (COREQ) instrument was used.

### Study setting

The research’s institutional context was at a federal public university, covering the health and social assistance departments of its four campuses, located in different municipalities in the countryside of the state of São Paulo, Brazil. In these departments, there are physical, material and professional resources aimed at serving the university community, through the provision of services focused on health promotion, prevention and treatment of injuries and social issues, including mental health actions and provision of sports and leisure activities, especially, aiming to ensure equity in opportunities in relation to the exercise of academic activities.

### Data source

The action research was carried out in 2020, based on the model by Werneck, Faria, Campos^(9)^, and, according to its premises, a collective work was developed consisting of three steps, with flexibility, in order to ensure the active participation of all components of the teams involved with the health problem in focus, based on the professional specificities of each one and on the interfaces between them, with an agreement on responsibilities in protocol production and execution.

In step 1, in order to contribute to “problem characterization”, psychological distress of undergraduate students from the four campuses, a descriptive and retrospective observational study was carried out on the demographic, education and health care profile of students assisted in the health units of each campus and in the university as a whole.

Step 2 corresponded to the intervention itself, together with 33 professionals involved in the management and care of students’ health and social assistance, 8 social workers, 6 psychologists, 6 doctors, 5 nurses, 2 nursing technicians, 1 therapist occupational therapist, 1 physical educator, 1 administrator and 3 administrative assistants. The intervention aimed to collectively elaborate the protocol, considering the action research premises, step 1 results and the components of the adopted model. Thus, “problem characterization” was complemented, establishing the protocol’s objective, its “plan of action”, respective responsible parties and “monitoring and assessment mechanisms”.

Step 3 consisted of finalizing and validating the protocol prepared by all its authors and forwarding its final version for approval and institutional adoption.

### Data collection and organization

To start work, previously, the management’ and participants’ consent was requested to carry out the research, obtaining approval. This research project was approved in accordance with the national and international ethics guidelines by the Research Ethics Committee of the *Universidade Estadual Paulista* (UNESP), whose opinion is attached to this submission. All formal procedures were made official, with signing the Informed Consent Form (ICF) online, for professionals to participate in the research, with the waiver of signing such term being authorized by students, participants of the first step.

In step 1, the study was carried out considering a probabilistic and stratified sample of students with complaints related to psychological distress among all students attended, in 2019, in the health units of the four university campuses. To establish the sample, initially, the number of undergraduate students assisted by nurses, physicians, psychologists and occupational therapists on each campus in that year was surveyed. Through a search in the physical records of these students, those who underwent consultation with these professionals due to problems related to mental health were identified.

The collection of data from the physical records of students included in the sample occurred after drawing lots and was guided by a previously designed instrument consisting of 12 items divided into: identification; age; place of birth; date of birth; sex; course; course year; color/race; complaint related to psychological distress; nursing diagnoses and/or those of other health professionals and the professional who provided care; ICD-10 and professional who performed the service.

The analysis of these data was carried out based on descriptive statistics, being presented according to the studied variables’ relative and absolute frequency (n and %), with the support of Excel and SPSS 13.0 (Statistical Package for the Social Sciences), version 2019, programs.

The intervention carried out in step 2 consisted of seven synchronous meetings of approximately two hours each and several asynchronous moments. During the synchronous meetings, initially, an attempt was made to deepen and discuss the theoretical and practical aspects of approaching psychological distress in undergraduate students, its causes and its impact on students and the university. Also, conceptual alignment was promoted among participants on health care protocols, especially service organization^(9)^. Next, the results of the observational study carried out in step 1 were presented.

Concomitantly, to complement “problem characterization”, a survey was carried out using an electronic form on the determinants and effects of psychological distress on undergraduate students as well as on facilities and difficulties faced in approaching this problem, from professionals’ perspective. The results were also presented in a synchronous meeting with them, with the achievement of consensus on the definition of the protocol’s objective to standardize actions for coping with psychological distress.

In synchronous meetings and subsequent asynchronous activities, an “action plan” was elaborated corresponding to the achievement of the outlined objective, which included the systematization of viable and pertinent activities to face the problem in question to be complemented and/or implemented at the university and which professional categories are involved in this implementation, with a description of the main aspects to make them viable and their “monitoring and assessment mechanisms”.

Thus, the protocol was constituted and agreed upon, with the proposal to reorganize the work process of health and social assistance professionals, by management representatives and those who provide assistance to students at the university institution under study.

After each synchronous meeting, there was an assessment by participants and consequent adaptation and adjustments of the next ones.

In step 3, in two synchronous meetings per campus, the protocol elaborated in the previous steps was presented, readjusted and validated by the professionals involved with its elaboration, in order to follow the institutional procedures for its approval.

### Results of work steps

The study carried out in step 1 identified that, in 2019, 8,267 clinical consultations were carried out by professionals from different categories, in the university’s health units, for 4,812 students: 552 on campus A; 253 on campus B; 445 on campus C; and 3,562 on campus D. In that same year, a total of 610 students who presented symptoms/complaints related to psychological distress were computed: on campus A, of the total number of students assisted, 142 (25.7%) presented these symptoms/complaints; on campus B, 39 (15.4%); on campus C, 164 (36.8%), with detailed data on attendances as of March 2019; and on campus D, the data of those who had symptoms/complaints related to psychological distress referred to the mental health care service, carried out from May 2019, totaling 265 (7.4%) students who had these symptoms/complaints.

For the sample calculation, the prevalence of students with symptoms/complaints related to psychological distress found on each university campus was considered. Following statistical support guidelines, a prevalence rate of 35% was established, making it possible to delimit the sample size of 214 (100%) students to be studied, stratified into: 50 (23.4%) students from campus A; 14 (6.5%) students from campus B; 57 (26.6%) students from campus C; and 93 (43.5%) students from campus D.

Regarding the category of professionals who individually assisted students with symptoms/complaints of psychological distress, it was found that psychology was the most frequent, with psychologists assisting 143 (66.8%) students in 2019, followed by the medical and nursing category, which together accounted for 98 (45.8%) of students attended that same year.

Regarding demographic aspects, a higher frequency of females (135; 63.1%) and white (115; 53.7%) was found among the total number of students attended with symptoms/complaints of psychological distress. There was a higher frequency of those aged between 21 and 24 years old (89, 41.5%), followed by 17 to 20 years old (85, 39.7%).

As for undergraduate courses, of the 45 (100%) existing at the university, in 40 (89%) of them, students treated with symptoms/complaints of psychological distress were more frequently enrolled in the biological sciences course, 32 (15%), followed by courses in agronomic engineering, environmental engineering and administration, with 13 (6.1%) in each. Regarding the course year in which the students were enrolled, the first year stood out, with 66 (30.8%). There were 57 (26.6%) in the second year, 37 (17.3%) in the third year, and 38 (17.8%) in the fourth year.

Students’ symptoms/complaints related to psychological distress were diverse, with a variation of 70 identified. Anxiety was the most frequent symptom (43.9%), followed by difficulties in dealing with family problems (26.6%), relationship difficulties (21.5%), insomnia (19.6%) and difficulty/excess with academic activities (16.4%).

In step 2, the intervention participants pointed out as determinants of psychological distress in undergraduate students: difficulties in adapting to university life, in academic performance, in dealing with one’s own autonomy and with relationships and in accepting negative; excessive academic tasks/academic pressure; absence of routine; bullying; moral harassment; estrangement from the family or excessive family protection; changing roles; cultural changes/differences; charge of society; isolation/social distancing; psychic immaturity; frustration of expectations; immediacy; extreme perfectionism; elevated anxiety; pre-existing illnesses; mental disorders; use of medication and psychotherapeutic follow-up before admission; use of illicit substances; various vulnerabilities; and low socioeconomic conditions.

As effects of this injury for students, professionals indicated: illness; acute suffering; greater aggressiveness; insecurity; depression; loneliness; increased frustration; feeling of incapacity; low self-esteem; difficulty in self-acceptance; functional impairment and impairment in interpersonal relationships; isolation; family distancing; difficulty with academic requirements; withdrawal from the course; truancy; vulnerability to mental illness; harmful behaviors; high attempts at control; abusive and harmful use of alcohol and other drugs; difficulty socializing; and suicidal behavior. And as effects of students’ psychological distress for the university, professionals mentioned: high failure rate; course retention; vacancy loss; evasion; need for team-oriented care; work overload; need to update to meet needs; and adequacy of services.

The professionals listed as facilities for approaching undergraduate students’ psychological distress the affirmative actions and student permanence already carried out within the university. Among the difficulties, they pointed out the lack of articulation between sectors and departments, information systems that need to be updated, problems with the physical structure that promotes health, insufficient hiring of human resources and lack of technical-organizational direction for approaching students’ psychological distress in the school context, with a lack of understanding and collaboration from other sectors from the university. They also considered that many problems are found in the academic sphere, such as student-professor ratio, overload of student tasks and also the lack of compliance of students in collective activities, limiting the possibility of action by professionals. It is noteworthy that, in the survey of facilities and difficulties for coping with students’ psychological distress, there was convergence among the research participants in recognizing the need to ensure, in a protocol and agreed way, intra and inter-institutionally, the due reception, adequate service and referral, with the possibility of solving the problem in question.

Still in step 2, regarding the “action plan”, a list of possible actions was established to face this grievance in focus, which were classified into collective activities through the holding of discussion groups, workshops and conversation circles, which were subdivided into three fronts, these being of an educational nature with students, therapeutic and support with students and permanent education in health for professionals of the four campuses. Individual activities were classified as therapeutic and supportive with students, also including cultural and leisure activities as well as physical and sports activities.

In step 3, the possible reach of the protocol’s objective was checked, which has as its main axis the reduction of psychological distress in undergraduate students, through proposed actions and other components as well as validation by its authors, both the form of publication and its content.

## DISCUSSION

It is considered that the process for producing the protocol was scientifically supported and methodologically guided by action research^(8)^ principles and by the proposed model by Werneck, Faria, Campos^(9)^, with the broad and active participation of the professionals involved, from start to finish, in line with the Unified Health System (SUS - *Sistema Único de Saúde*), Unified Social Assistance System (SUAS - *Sistema Único de Assistência Social*) and institutional policy premises, especially aimed at promoting students’ mental health and the appropriate approach to undergraduate students’ psychological distress.

For “problem characterization: undergraduate students’ psychological distress”, during the intervention in conjunction with professionals from the four campuses of the aforementioned university, it was possible to give visibility to the demographic, training and health care profiles of students assisted in the units of this institution, especially the profiles of those with symptoms/complaints related to psychological distress.

During these meetings, it was also possible to list aspects considered by participants as determinants and implications for students and the university in relation to psychological distress, in addition to the facilities and difficulties related to the daily work of health care for students.

At the studied university, several support fronts for students have been developed, which should be added to the activities to be implemented, according to the proposal of the elaborated protocol. Among the existing activities, professionals highlighted the front of student permanence for scholarship students through the Student Monitoring Program, which is a systematized process of diverse actions, organized in a network regarding issues that impact academic trajectories, offering resources and support for training university^(10)^ as well as allowing periodic meetings of professionals from each campus and multicampus, for assessing the actions carried out and reflection on those that should be implemented, discussion of cases and possibilities of intra and extra institutional referrals. It should be noted that, for non-scholarship students, the same health care and support services are offered.

It was found, however, that professionals find obstacles to perform an effective service, especially in relation to the reduced team and the non-involvement of the university’s professionals to effectively recognize and face psychological distress. It is postulated that university management should pay close attention to key points that need to be rethought and discussed, both regarding physical infrastructure and organizational aspects and human resource quantity and qualification^(3,6)^.

While recognizing the great potential of the work carried out in the health units of the university’s four campuses to approach its students’ mental health, composing the psychosocial health care network of the different host municipalities, the professionals participating in this action research expressed many weaknesses in the referred networks’ structure and lack of legitimacy for professionals from other areas that are not exclusive to metal health. It was evident, through the discussions carried out in the synchronous meetings, that it is necessary to strengthen the relations with the networks, in order to favor the service of reference and counter-reference, seeking comprehensiveness for students’ health care.

Based on these data, prepared and discussed by the intervention participants themselves, it was possible to measure the magnitude, transcendence, vulnerability and determinants of psychological distress for students, relating them to their implications for them and for the university, bringing subsidies for “action plan” discussion and proposition.

In this sense, the elaborated protocol consisted of a wide range of proposed activities based on scientific and interdisciplinary knowledge and on concrete institutional conditions to be successfully implemented on the four campuses, provided that there is support from management and co-responsibility of professionals for such. It is added that the protocol, together with the other actions, was indicated by all participants as a fundamental resource to promote/strengthen health actions aimed at coping with psychological distress.

It is considered that carrying out action research and its product constituted an appropriate strategy for the promotion of mental health actions that allow developing students’ cognitive, social, cultural and emotional, enabling open spaces for dialogue and listening in a place where they have the opportunity to speak and express themselves so that they can perform movements to re-signify themselves, the other and their context^(1)^. Furthermore, it was reaffirmed that the university should promote actions for mental health problem prevention and treatment, such as psychological distress, in order to enable adaptations to real training needs, without compromising undergraduate students’ cognitive and socio-emotional skills^(2-3)^. As another positive aspect of this experience, there was a strengthening of professionals’ role in conducting planning, taking advantage of information production and knowledge application and strengthening existing relationships between the organization and its base through participatory methods^(8)^.

However, it was found that at no time was the inclusion of students themselves and/or their representation in elaboration/review of the protocol under construction considered by the research participants, in order to expand their qualification possibilities. At the same time, it was noted that there was little appreciation of action propositions that included families as the focus of actions, even considering that family problems are the second most prevalent cause of symptoms/complaints related to the occurrence of psychological distress among undergraduate students at the institution, aspects that need to be taken up again in future opportunities to review this protocol.

### Study limitations

Carrying out this research amidst the COVID-19 pandemic prevented the face-to-face participation of professionals in the proposed intervention, with many absences in synchronous virtual meetings justified due to this disease or the overload it brought. For the same reason, it is accepted as a limitation the infeasibility of the participation of students or their representatives, because they are in social distance, with academic activities suspended throughout fieldwork.

### Contributions to health

It is believed that, with the standardization of activities based on the elaborated protocol, it will become possible to reorient the work process in the health and social assistance departments of the university institution studied, enabling, after its implementation, the production of positive impacts on students’ quality of life and health, its main focus, with possible results that will ultimately favor the health, education and university management professionals involved. More broadly, it is expected that the experience reported here can contribute to encourage and support other university institutions to implement actions to cope with their students’ psychological distress.

## FINAL CONSIDERATIONS

The service organization protocol was an essential management tool both to qualify the actions already developed with a view to promoting mental health, preventing psychological distress and assisting undergraduate students in the face of this problem, and for implementing new actions with the same purposes. Moreover, during this course, there was an opportunity to establish a rapprochement between management and social assistance and health professionals intra and inter campus, facilitating the exchange of their conceptions, visions and experiences in the area, and strengthening the chances of success in the tackling the problem at hand.

Finally, recognizing that a care protocol, after being designed and implemented, has transitory validity, its periodic assessment and consequent modification are recommended, considering the circumstances involved, the operational capacity and the current epidemiological profile as well as the essential condition that these activities are carried out collectively by their authors and other professionals who may be involved in their application in the future, in addition to including students’ active participation in this process.
